# Improving the Completion of Capacity and Consent Assessments

**DOI:** 10.1192/bjo.2024.94

**Published:** 2024-08-01

**Authors:** Michael Abbott, Kristina Logacheva, Eleanor Thompson, Nik Fikri bin Nik Fauzi, Tarun Rangan

**Affiliations:** 1 South West London and St George's Mental Health NHS Trust, London, United Kingdom; 2 Barnet, Enfield and Haringey Mental Health NHS Trust, London, United Kingdom

## Abstract

**Aims:**

The respect of a patient's autonomy and choices is one of the cornerstones of good psychiatric care. Key to this is ensuring the timely and thorough assessment of a patient's capacity to consent to admission and treatment once in contact with mental health services.

The aim of this quality improvement project was to optimise the Trustwide completion of capacity assessments for all newly admitted patients within 24 hours of admission at South West London and St Georges Mental Health NHS Trust. Our goal was for 100% completion of the Trust's Brief Capacity and Consent (BCAC) form by July 2023.

**Methods:**

We obtained a list of all new admissions to inpatient wards across the Trust's three hospital sites between 1–14 February 2023. A retrospective audit was then undertaken to establish the baseline BCAC completion rate. Following this a quality improvement strategy was created using the Plan-Do-Study-Act (PDSA) model of creating change. A series of three interventions were implemented and subsequently analysed using the PDSA model. These included creating an admission checklist and placing this in doctors' on-call rooms across the trust, distributing the checklist to wards across hospital sites and service lines and lastly, sending out reminder emails to doctors at the Trust. A re-audit was completed after each intervention.

**Results:**

The baseline BCAC completion rate was 60.5% (n = 76) in February 2023. This increased to 63.75% (n = 80) following the creation of the admission checklist. Distributing the checklist throughout the Trust resulted in a further improvement to 76.5% (n = 81). In July 2023, the final data collected 6 weeks post reminder email demonstrated a BCAC completion rate of 75% (n = 68).

**Conclusion:**

Through the application of the PDSA model, alongside focused interventions aimed at raising Trust-wide awareness of the BCAC form, we managed to obtain a 14.5% increase in BCAC completion rates between 13 February and 31 July 2023. Whilst not achieving the initial goal of 100% completion, this quality improvement project helped to bring a renewed focus to the timely completion of capacity and consent assessments at the Trust. Moving forward, we hope to build on this improvement by exploring additional strategies to reach a 100% BCAC completion rate, thereby promoting patient safety and autonomy.

